# Hand-Book for the Hospital Corps of the U. S. Army and State Military Forces

**Published:** 1899-07

**Authors:** 


### Books and Magazines.

HAND-BOOK for the Hospital Corps of the U. S. Army and State Military Forces. By Chas. Smart, Deputy Surgeon General U. S. A. Approved by the Surgeon General of the Army. Published by William Wood and Company, New York. 1898. Pages, 340. Price, cloth $2.25.

This is the official text-book of army medical regulations. The first 38 pages are devoted to hospital discipline and organization. Sixty pages are then devoted to camp sanitation, and in this connection it is to be regretted that the excellent principles and advice contained in them were not observed in the late war. Great attention in detail is given to general and special sanitary science throughout. The latter part of the work is a complete treatise on first aid, and the best one it has ever been my pleasure to read. The work is worth its price ten times over to any physician or nurse. It was rewritten after the war with Spain was a certainty, for the purpose, as set forth, of raising the standard of efficiency in all branches of the service, and of giving the enlisted men a practical guide for individual hygiene. I. J. J.

